# Infrared Ship Detection in Complex Nearshore Scenes Based on Improved YOLOv5s

**DOI:** 10.3390/s25133979

**Published:** 2025-06-26

**Authors:** Xiuwen Liu, Mingchen Liu, Yong Yin

**Affiliations:** Key Laboratory of Marine Simulation and Control, Navigation College, Dalian Maritime University, Dalian 116026, China; lmc0024@dlmu.edu.cn (M.L.); bushyin@dlmu.edu.cn (Y.Y.)

**Keywords:** nearshore waters, ship detection, infrared vision, YOLOv5s

## Abstract

Ensuring navigational safety in nearshore waters is essential for the sustainable development of the shipping economy. Accurate ship identification and classification are central to this objective, underscoring the critical importance of ship detection technology. However, compared to open-sea surface, dense vessel distributions and complex backgrounds in nearshore areas substantially limit detection efficacy. Infrared vision sensors offer distinct advantages over visible light by enabling reliable target detection in all weather conditions. This study therefore proposes CGSE-YOLOv5s, an enhanced YOLOv5s-based algorithm specifically designed for complex infrared nearshore scenarios. Three key improvements are introduced: (1) Contrast Limited Adaptive Histogram Equalization integrated with Gaussian Filtering enhances target edge sharpness; (2) Replacement of the feature pyramid network’s C3 module with a Swin Transformer-based C3STR module reduces multi-scale false detections; and (3) Implementation of an Efficient Channel Attention mechanism amplifies critical target features. Experimental results demonstrate that CGSE-YOLOv5s achieves a mean average precision (mAP@0.5) of 94.8%, outperforming YOLOv5s by 1.3% and surpassing other detection algorithms.

## 1. Introduction

Shipping is an important link in the global trade network. With the continuous expansion of the scale of international trade, the flow of ships presents significant growth, with this development trend being particularly prominent in major ports and nearshore waters. Furthermore, this growth creates higher requirements regarding the safety of navigation in nearshore waters [1]. Unlike the open sea, the traffic environment in nearshore waters is more complex, featuring narrow waterways and large numbers of ships gathered at ports. There are many types of ships, which present great differences in their speed and maneuverability; therefore, if there is no timely warning regarding dangerous situations, collisions between ships, ship–bridge collisions, and other major traffic accidents may occur, especially at night or under poor visibility conditions. In this context, the performance of target detection methods directly affects the safety of navigation. Therefore, it is of great practical significance to accurately realize ship detection in complex nearshore scenarios.

At present, sensor technologies applied in the ship detection field are based on four main categories: remote sensing image-, navigation radar-, visible light vision-, and infrared vision-based detection. Satellite remote sensing imagery is known for its wide field of view, high point of view, and fast data acquisition capability; however, it is not applicable to real-time detection due to the delayed signal transmission and the relatively low resolution of the image, making it inapplicable for the accurate detection of ships [2,3,4]. Navigation radar has the advantage of facilitating all-weather detection; however, targets are presented as points or bands, making it difficult to recognize ship scale and category information [5,6,7]. Detection based on visible light is characterized by high resolution and allows rich target feature information to be obtained; however, it is greatly affected by the environment, and it is difficult to distinguish targets at night or in bad weather conditions, in which case the detection effect will be seriously affected [8,9,10]. Regarding detection based on infrared vision, the resolution of imagery acquired using infrared sensors has a certain gap in comparison with visible light sensors; however, its imaging mode is based on the infrared radiation emitted by the object itself, and passive detection can be realized by capturing the characteristic temperature differences between the target and the background; thus, it has a natural sensitivity to target with heat sources (e.g., ship’s engine, personnel body temperature). In this way, it can effectively identify targets according to their heat signal, while the background sea surface forms a stable low-temperature field, significantly contrasting with the target [11,12,13]. Therefore, infrared vision allows for the acquisition of environmental information around the clock, providing ideal application conditions for ship detection in complex nearshore scenarios.

Infrared target detection algorithms can be macroscopically categorized into two groups: traditional detection algorithms and deep learning-based detection algorithms. In terms of traditional algorithms, scholars have carried out a great deal of research in the past. For example, Pokrajac et al. [14] effectively detected moving objects in infrared videos by suppressing the effects of background noise based on the dimensionality reduction decomposition of spatio-temporal blocks. Man et al. [15] constructed an infrared single-frame detection method by extracting blocks from images and finding similar blocks, which achieved good detection results in the infrared small target detection task under complex background interference. Khare et al. [16] have proposed a background modeling method to achieve target detection using statistical changes in infrared pixels. Liu et al. [17] have proposed a non-convex tensor low-rank approximation (NTLA) method for the detection of small infrared targets, which achieved accurate background estimation in complex scenes. Although the above traditional detection algorithms have achieved certain results, they rely on the separation of background and target in the image, and suffer from defects relating to limited feature extraction and biased classification ability, leading to insufficient generalization ability to different infrared scenes. In recent years, the rapid progress of deep learning technology has greatly promoted the development of target detection research. Convolutional neural networks, with their excellent feature learning ability, not only simplify the complex process of artificial feature design but can also effectively focus on the target itself, filtering out background interference. Therefore, the computational accuracy and generalization ability in the field of target detection have been significantly improved, successfully addressing many problems faced by traditional target detection approaches. Deep learning-based detection algorithms can be mainly categorized into two types: two-stage target detection models, represented by the R-CNN [18,19] series of algorithms; and single-stage target detection models, represented by the YOLO [20] series of algorithms. In the former, target detection is accomplished using a two-stage algorithm that first extracts the candidate region and then classifies and recognizes the region; these algorithms possess high accuracy but exhibit poor real-time performance due to a large number of complex calculations. Compared to two-stage algorithms, the single-stage algorithms skip the candidate region extraction phase and directly generate the category probability and position coordinate values of objects, significantly improving the speed in the target detection task. For example, Firdiantika et al. [21] applied the CA attention mechanism to a single-stage network, which worked well for the detection of small targets. Zhang et al. [22] have proposed a YOLO-IR-Free method based on an anchorless detection head, which simplifies the process of anchor selection and adjustment, optimizes the convergence speed of the model, and better achieves real-time detection. Bao et al. [23] designed a dual feature extraction channel based on YOLO for infrared and visible images. This method compensates for the lack of target texture features in infrared images, unifies infrared and visible features, and reduces the impact of redundant information on the accuracy of target detection. Zhang et al. [24] developed an efficient lightweight convolutional neural network and designed a new bi-directional weighted feature pyramid network (BWFPN) by integrating multi-scale features. This strategy not only improves the detection accuracy of the model but also ensures the computational efficiency of the model. Li et al. [25] have proposed an improved YOLO-FIR algorithm, in which the CSP module in the shallow layer is extended and iterated in the feature extraction part, the improved SK attention module is invoked to improve the robustness of the model, and the structure of the network detection head is improved, which significantly increases the detection accuracy of small targets in infrared imagery.

The YOLO algorithms possess unique performance advantages for application to infrared scenes; however, in the existing literature, it can be found that most deep learning-based infrared target detection research has focused on unilateral optimization of the network structure and, although such methods can achieve good results in target detection in some fields, there are still unsolved problems regarding the detection of ships in infrared imagery considering the characteristics of nearshore scenes. In recent years, some scholars have already conducted relevant research in the field of infrared ship detection using deep learning methods. For example, Liu et al. [26] proposed a new method of MAFF-DETR with the advantage of detecting small infrared ship targets, but the method could not solve the missed detection behavior in the case of severe target occlusion. Wang et al. [27] optimized the YOLOv5 algorithm by combining the PP-LCNet backbone network with it to design a lightweight detection algorithm, which effectively reduces the computational load of the model at the expense of some of the detection performance in the complex context of the nearshore.

However, by analyzing the imaging characteristics of infrared technology and the distribution characteristics of nearshore vessels, we believe that the challenges faced in the detection process still exist as follows: (1) compared with visible light, the captured infrared image will have insufficient resolution, resulting in blurring of detailed features of the ship, and there are a variety of backgrounds such as vegetation and rocks near the coastline that exhibit temperature, texture, and other characteristics of infrared radiation, which are easy to confuse with the ship’s target; furthermore, the blurring of the features and confusion often lead to false detection of the target. (2) Compared with imagery captured in the far sea direction, the high density of nearshore ships and the intensive parking of ships in the harbor area lead to problems such as mutual obscuration between ships and ships, ships and buildings, and ships and terminal facilities, which will lead to the absence of some features of the ship and increase the difficulty of detection. (3) Various types of ships make significant size differences between ships, which leads to the complex background of the acquired images and makes it difficult to achieve the expected detection results, which puts higher requirements on the detection effectiveness of multi-scale ships. Therefore, when applying deep learning-based target detection algorithms to nearshore ship detection in complex scenes, they should be optimized for the improvement of infrared image quality, the focus on key features, and the enhancement of multiscale detection performance, to provide better conditions for the realization of accurate ship detection.

To address the above problems, this study proposes an algorithm (called CGSE-YOLOv5s) for the ship target detection task in complex nearshore scenes. The contributions of this paper are summarized as follows:

- We propose a new method for detecting ship targets in nearshore waters. The approach involves enhancing the infrared image and optimizing the network structure separately, then combining them to improve the ship detection performance in complex nearshore scenes.
- We perform infrared image enhancement by combining Contrast Limited Adaptive Histogram Equalization with Gaussian Filter (CLAHE-GF), apply it to the input layer of the model, and compare the practical effectiveness of different data enhancement methods.
- We integrate the Swin Transformer coding structure into the C3 module of the neck layer and verify its effect in terms of model performance improvement.
- We introduce the Efficient Channel Attention (ECA) mechanism and integrate it in front of each detection head, additionally comparing the impacts of different attention mechanisms on model performance.
- We compare the enhancement effect of the improved model with respect to the traditional model.
- We add and replace the improvement modules from different architectures to compare their effects on the proposed model.
- We use the same infrared nearshore vessel dataset to compare the proposed model with several other target detection models.
- Our experimental results show that the method proposed in this study exhibits excellent performance in the nearshore ship detection task.

The remaining sections of the paper are structured as follows: Section 2 details CGSE-YOLOv5s; Section 3 presents the dataset and the various experiments; Section 4 discusses the results; Section 5 concludes the paper.

## 2. Proposed Approach

### 2.1. Overview of YOLOv5s

**Figure 1:**
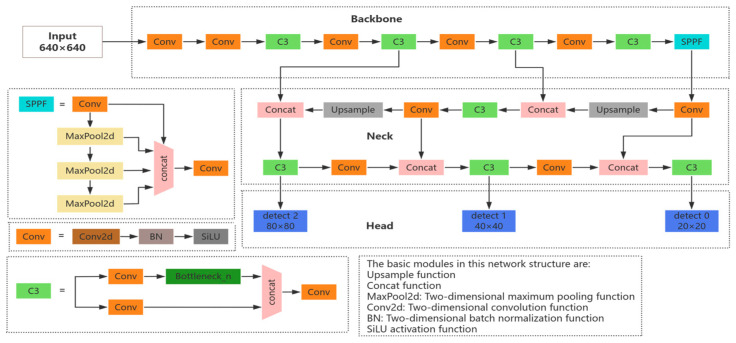
Structure of the YOLOv5s network.

There are a total of four versions—namely, YOLOv5s, YOLOv5m, YOLOv5l, and YOLOv5x—in the YOLOv5 [28] target detection network. Of these, YOLOv5s has the smallest depth and feature map width. Therefore, in order to realize a lightweight model and better meet the real-time requirements of the considered task, we chose YOLOv5s as the baseline network for this experiment. The YOLOv5s network structure is mainly composed of Input, Backbone, Neck, and Head modules, as shown in Figure 1.

The Input layer data are processed via Mosaic data augmentation, adaptive image computation, and adaptive anchor frame computation. Mosaic data augmentation involves randomly splicing multiple images to enable the model to learn the features of locally cropped targets with different backgrounds, in order to alleviate the model’s overfitting to the target’s location, scale, and background; meanwhile, adaptive image computation reduces the computational burden by uniformly scaling the input images to a resolution of 640 × 640, maintaining the original aspect ratio while minimizing redundant computations. Adaptive Anchor Frame Computing automatically optimizes the size of the preset anchor frame (Anchor) based on the statistical properties of the training dataset, in order to reduce dependence on manual adjustments.

The Backbone network mainly consists of Conv (CBS), C3, and SPPF (Spatial Pyramid Pooling) modules that extract features and continuously reduce the feature map by stacking convolutional layers with downsampling operations, in which the CBS module consists of a 2D convolutional function, a 2D batch normalization layer, and a SiLU activation function; the C3 module consists of three CBS modules and a Bottleneck module; and the SPPF consists of three 5 × 5 Maxpool2d convolutional kernels for serial computation.

The Neck network adopts the combined structure of feature pyramid (FPN) [29] and path aggregation (PAN) [30], which first fuses the feature maps in a level-by-level manner by passing semantic information from the top down, in order to improve the detection accuracy for large targets, and then performs further fusion to improve the small target detection accuracy by passing localization information from bottom up. The main role of this part is to mix shallow features extracted from the Backbone with deep semantic features, thus realizing multi-scale target detection.

The Head layer includes a Detect module with three detection feature layers, which performs prediction at the 80 × 80, 40 × 40, and 20 × 20 scales, corresponding to anchor frames of different sizes in order to better adapt to different sizes of targets. This layer is responsible for the final regression prediction.

### 2.2. Input Layer Infrared Image Enhancement

In complex nearshore scenes, the data collected using infrared technology have the following characteristics: (1) the image pixels are not uniformly distributed; (2) the target texture information within the image is not obvious; and (3) the target is susceptible to interference from background noise. In order to enhance the distinction between the target and background and suppress background noise, thus providing richer semantic information for subsequent detection algorithms, we adopt the Contrast Limited Adaptive Histogram Equalization (CLAHE) [31] combined with Gaussian Filtering (GF) approach to enhance infrared images in the Input layer.

#### 2.2.1. Contrast Limited Adaptive Histogram Equalization

Compared with visible light images, infrared images usually suffer from the problems of background–target confusion and insufficient target texture information. In order to solve this problem, we use a CLAHE algorithm to obtain more image details, with the aim of improving the quality of the infrared images and providing more effective feature information in the subsequent training of the model. The commonly used Adaptive Histogram Equalization (AHE) [32] algorithm divides the image region into numerous localized cell blocks, performs corresponding histogram equalization for each block, and finally redistributes the luminance, which alters the image’s contrast by mapping the pixel values of localized low-contrast regions in the original image into a wider range; however, the algorithm can over-amplify small amounts of noise in flat regions with close pixel values. The CLAHE algorithm is optimized on the basis of the AHE algorithm. In CLAHE, the pixels within each chunk are divided into two parts: low-contrast pixels and high-contrast pixels. Low-contrast pixels are given smaller weights, while high-contrast pixels are given larger weights. These weights are then used to calculate a cumulative histogram for the small block, and the value of each pixel is adjusted based on that cumulative histogram. This adjustment results in smaller changes in the low-contrast pixels and larger changes in the high-contrast pixels, thus improving the contrast of the image. Equations (1) to (5) explain the adjustment process of the histogram height of the local area. This method enhances the local contrast while controlling the disadvantage of too much background noise, thus effectively addressing the problem of blurred infrared pixels.

(1) Calculate the local mapping function and set the sliding block with the size of *M × M* (we use 16 × 16):

(1) $$m(i)=\frac{255\times C_{DF}(i)}{M\times M}$$

(2) Calculate the cumulative distribution function of the local histogram of the sliding block:

(2) $$S=\frac{d(m(i))}{di}=H_{ist}(i)\times \frac{255}{M\times M}$$

It can be seen from Equation (2) that when the height of the local histogram is limited, the slope of the mapping function can also be limited. The over-enhancement of the local contrast can be effectively suppressed in this way.

(3) Set the maximum slope to *S*_max_ to obtain the maximum height of the histogram:

(3) $$H_{\max}=S_{\max}\times \frac{255}{M\times M}$$

(4) Set the threshold value *T*, where *T* ≥ *H*_max_; truncate the part with histogram height greater than *H*_max_; then, distribute the truncated part evenly in the whole gray scale range of the image. In this way, the histogram height can be increased as a whole without changing the total area.

(5) The final improved histogram is shown in Equation (4):

(4) $$H_{ist}(i)=\left\{\begin{array}{l} H_{ist}(i)+L,H_{ist}(i)<T\\ H_{\max},H_{ist}(i)\geq T \end{array}\right.$$

**Figure 2:**
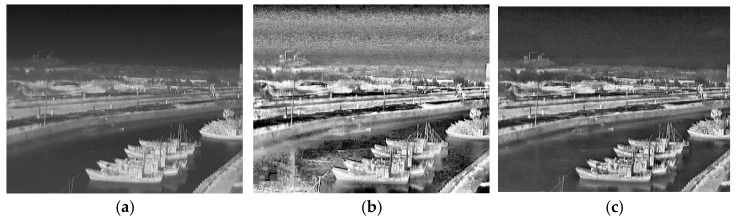
Comparison of images processed using the AHE and CLAHE algorithms. (**a**) Original image; (**b**) original + AHE; (**c**) original + CLAHE.

Figure 2a–c show a comparison between an unprocessed image and those processed using the AHE and CLAHE algorithms. The boundary between the ship and the background in the unprocessed image is relatively fuzzy, and the background noise is too strong in the image processed using the AHE algorithm. Meanwhile, the ship contour information within the image processed using the CLAHE algorithm is clearly visible, and there is a stronger differentiation between the target and the background.

#### 2.2.2. Gaussian Filtering Smoothing

**Figure 3:**
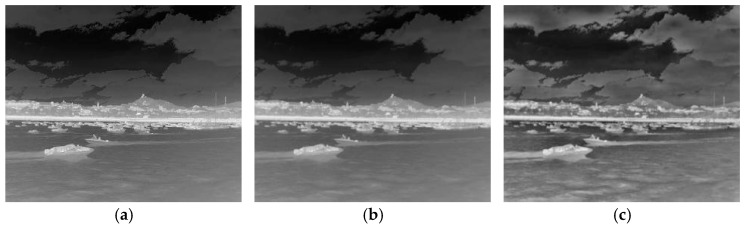
Comparison of infrared images after algorithmic enhancement. (**a**) Original image; (**b**) original + GF; (**c**) original + CLAHE + GF.

The core advantage of CLAHE is localized contrast enhancement, which is especially suitable for processing unevenly lit, low-contrast areas; however, in complex textured or flat areas, CLAHE-processed images may introduce blocking artifacts and amplify noise. Meanwhile, as nearshore waters are at the intersection of sea and land areas, the water mist and vapor generated by the temperature difference between land and water affect the infrared wave band due to atmospheric absorption and scattering, the acquired infrared imagery will also present various noise due to waves, ship currents, and wave light, which can result in serious interference in the ship detection task. GF is an image processing technique based on the principle of using a Gaussian function to perform two-dimensional convolution of an image to calculate the weighted average in the neighborhood around each pixel, thus achieving image smoothing [33]. As detailed in Equation (5), this method can effectively remove high-frequency noise from images while preserving edge and detail information. Therefore, based on the above analysis, we propose a strategy of sequential fusion of CLAHE and GF, using GF to suppress background clutter in the image processed by the CLAHE algorithm. The core idea is to take advantage of the complementary characteristics of the two, making the details of the image smoother and more natural without sacrificing the enhancement effect.

(5) $$I^{\prime }(x,y)=\frac{1}{2\pi \sigma^{2}}\iint I(u,v)e^{\left(-\frac{\left({\left(x-u\right)}^{2}+{\left(y-v\right)}^{2}\right)}{2\sigma^{2}}\right)}dudv$$

where *I′*(*x*,*y*) denotes the pixel value at point (*x*,*y*) in the output image; *I*(*u*,*v*) denotes the pixel value at point (*u*,*v*) in the input image; and σ is the standard deviation of the Gaussian kernel. The larger the Gaussian kernel, the larger the degree of smoothing, and we use a Gaussian kernel of size 11 × 11 in this study. A comparison of the effects of the algorithm-enhanced infrared images is shown in Figure 3. From Figure 3b, it can be seen that if only the infrared image is used for GF, although the background noise of the image can be greatly inhibited, it has a certain degree of impact on the target’s texture information within the image. From Figure 3c, it can be seen that the image obtained using the combination of the CLAHE and GF algorithms presents clear texture information in the target area while, at the same time, the interference due to invalid noise is eliminated.

### 2.3. C3STR Module

**Figure 4:**
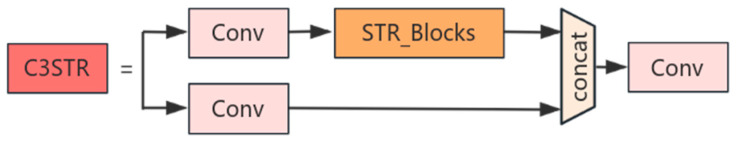
The structure of C3STR module.

The C3 module is a fundamental component of the YOLOv5 network architecture, typically characterized by a multi-branch structure that enables it to extract features at different levels. However, in practical ship detection scenarios, the scale of ship targets to be detected varies significantly, which poses challenges for traditional C3 modules in acquiring sufficient global information. In recent years, the emergence of Swin Transformer [34], which follows Vision Transformer (ViT) [35], is another important breakthrough in the field of computer vision. ViT achieves image feature modeling through the advantage of global self-attention, but this mechanism leads to a significant increase in computational cost, making it unsuitable for real-time processing tasks. Additionally, it can only output feature maps of a single scale, making it difficult to handle multi-scale targets. Compared with ViT, the core advantages of Swin Transformer lie in its Hierarchical Architecture and Shifted Window-based Self-Attention. The Hierarchical Architecture makes it more suitable for dense prediction tasks and enables efficient modeling of multi-scale information. The Shifted Window-based Self-Attention can effectively save computing resources and significantly enhance the robustness of multi-scale object detection. Swin Transformer has demonstrated excellent performance and application potential in computer vision tasks, with its prior application in fields such as medical image detection having already demonstrated impressive effects [36]. In recent years, the application of the Swin Transformer structure to the classification and inspection of ships has also broken new ground. Huang et al. [37] proposed for the first time a CNN-Swin model that enhances the focus on the overall ship architecture and scale features, and validated the effectiveness of the approach on a warship dataset. Inspired by the study, we used Swin Transformer blocks to create a new C3STR module, thus improving the leakage detection problem of small-scale ship targets in multi-scale ship scenes with complex backgrounds. As shown in Figure 4, the new C3STR module consists of Swin Transformer blocks replacing the Bottleneck in the original C3 module.

**Figure 5:**
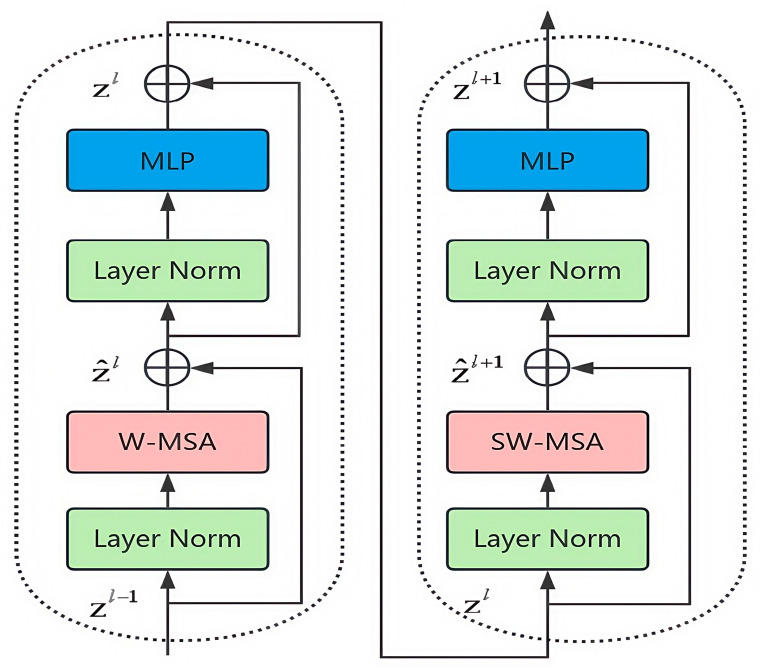
Two successive Swin Transformer blocks.

The Swin Transformer module mainly consists of a normalization layer (Layer Norm), a window self-attention module (W-MSA), a shifted-window self-attention module (SW-MSA), and a multilayer perceptron (MLP). The specific structure is shown in Figure 5. In particular, Layer Norm is used to normalize the input data, such that they are stable and easy to process; the W-MSA module is used to compute the self-attention inside the window; the SW-MSA module performs information transfer between neighboring windows by moving operations on the basis of W-MSA; and the MLP is used to integrate attentional information, extract features, and change the size of the image. It is worth noting that the W-MSA and SW-MSA modules can only integrate attention information into different windows and capture global features when used in combination.

The Swin Transformer module partitions the image into multiple non-overlapping windows of the same size and performs the self-attention operation in each window. Through the use of the non-shift and shift window partitioning scheme, the module not only reduces the amount of self-attention computations and the number of parameters, but can also pay full attention to the different regions within the image. In this way, it improves the perception ability regarding the geometric characteristics of small ship targets while ensuring high overall detection performance. The following formulas show the calculation process of this module:

(6) $${\hat{z}}^{l}=W-MSA(LN(z^{l-1}))+z^{l-1}$$

(7) $$z^{l}=MLP(LN({\hat{z}}^{l}))+{\hat{z}}^{l}$$

(8) $${\hat{z}}^{l+1}=SW-MSA(LN(z^{l}))+z^{l}$$

(9) $$z^{l+1}=MLP(LN({\hat{z}}^{l+1}))+{\hat{z}}^{l+1}$$

### 2.4. ECA Attention Mechanism

The background environment under nearshore conditions is intricate and complex, and the challenges faced include mutual occlusion between vessels and interference from external structures (such as clusters of buildings near the wharf), which can significantly reduce detection accuracy. Integrating the ECA mechanism into the Convolutional Neural Network (CNN) addresses this issue by dynamically adjusting feature weights [38]. This enhanced functionality enables the network to focus its attention on task-critical regions and channels, suppress irrelevant information and background noise, and simultaneously improve target localization capabilities, thereby enhancing model performance in multiple dimensions.

The ECA [39] mechanism is an improved version of the Squeeze-and-Excitation (SE) [40] attention mechanism, which effectively compensates for the defects of the dimensionality reduction process in the traditional SE module. The SE module uses fully connected layer dimensionality reduction in channel compression, which may lead to a loss of channel information, especially in small networks where aggressive compression can degrade the integrity of features. Compared to the SE module, the ECA module weights the features in different channels, which enables the network to better capture long-distance dependencies and increase the network’s focus on critical information.

**Figure 6:**
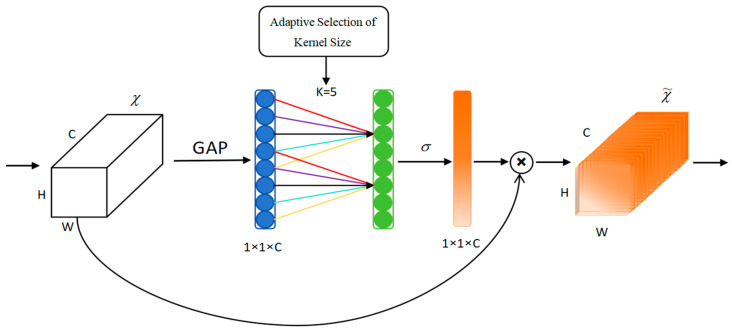
The structure of ECA module.

The ECA module initiates feature processing by applying global average pooling to input feature maps, subsequently utilizing a 1D convolution (with kernel size *k*) instead of traditional fully connected operations. This architecture preserves dimensional integrity while establishing efficient cross-channel communication through the use of weight-sharing mechanisms. Channel-specific weights *ω* are generated via sigmoid activation (Equation (10)), with the convolutional kernel size *k* being dynamically determined based on feature map channel count (Equation (11)), enabling more frequent cross-channel interactions in high-dimensional layers. The module completes its operation by performing element-wise multiplication between the computed weights and original features, followed by feature map updating and output generation (as illustrated in Figure 6). By integrating the ECA attention mechanism in front of the network detection head of YOLOv5s, we are able to adaptively calibrate the feature maps in different layers, which helps to improve the detection of ships in complex backgrounds.

(10) $$\omega =\sigma (C1D_{k}(y))$$

where *ω* is the channel weight, *σ* is the sigmoid activation function, *C1D* represents one-dimensional convolution, and *k* is the size of convolution kernel.

(11) $$k={\left|\frac{\log_{2}c}{\gamma }+\frac{b}{\gamma }\right|}_{odd}$$

where *γ* = 2, *b* = 1, *c* represents the size of the number of channels, and ||*_odd_* means that the generated convolution kernel size can only be an odd number.

### 2.5. The CGSE-YOLOv5s Detection Model

**Figure 7:**
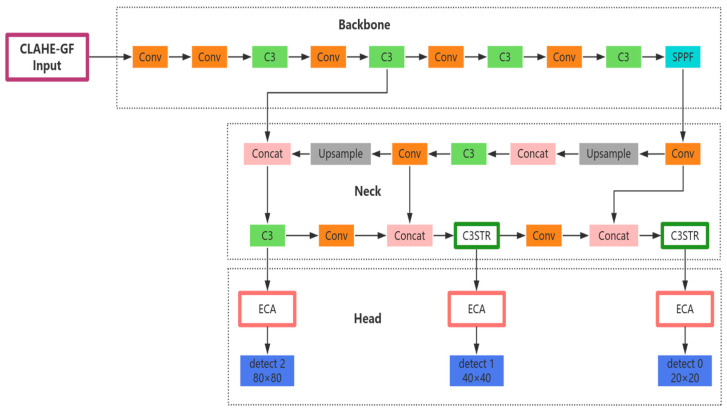
Structure of the CGSE-YOLOv5s network.

The distribution of ships in nearshore waters is usually characterized by agglomeration, as ships with diversified types and high densities not only berth at nearshore mooring areas but also overlap and block each other due to the belt-shaped distribution of ships along the fairway. The difficulty of detecting ships increases in such a complex scenario, and it is difficult to keep up with the detection requirements when traditional YOLOv5s algorithms are applied to actual scenarios in this context. Therefore, in order to improve the effectiveness of ship detection in nearshore waters, we propose an improved algorithm based on the YOLOv5s model called CGSE-YOLOv5s, which is a ship target detection algorithm designed specifically for infrared nearshore complex scenes; its overall structure is shown in Figure 7. In this study, to address the problems related to the insufficient resolution of infrared images and high background noise in nearshore waters, we enhance these images based on a hybrid CLAHE-GF pre-processing strategy in order to improve the quality of the images in the model input stage. Aiming at the problem of false detection and misdetection of ships due to large differences in ship scales, the Swin Transformer’s hierarchical self-attention approach is used to replace the C3 module in the PAN structure of the feature fusion network (Neck layer) with the C3STR module, in order to improve the efficiency and accuracy of feature extraction and fusion, thus enhancing the ability of the model to detect multi-scale targets. In order to alleviate the problem of mutual overlapping and occlusion of ships, we construct the ECA-Head structure by embedding the ECA attention mechanism in the detection head—in particular, front-loading each detection branch—in order to strengthen the model’s attention to key ship features, thus improving the model’s detection performance.

## 3. Experiment and Result Analysis

### 3.1. Experimental Dataset

**Figure 8:**
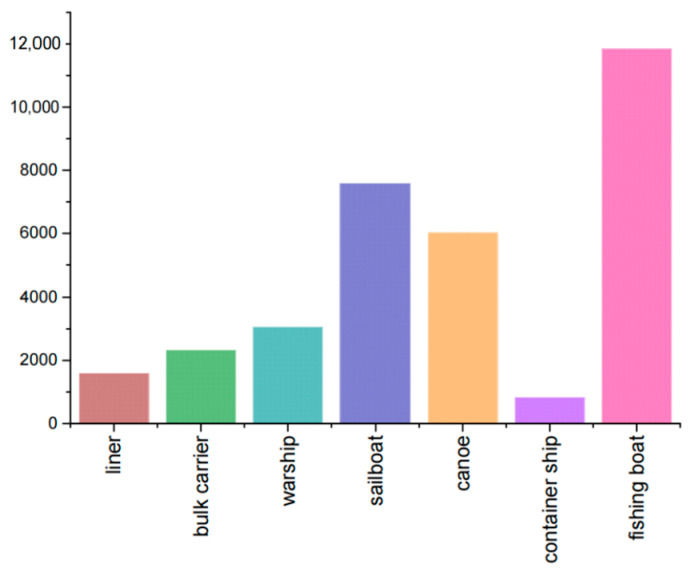
Summary of ship labels in the dataset.

The dataset used in this experiment for training and testing the improved model was mainly sourced from the InfiRay Infrared Open Source Database [41], which is an infrared image dataset for target detection of ships at sea, in addition to 977 infrared images collected at several ports and terminals in Dalian, China, which were used to supplement the nearshore scenario data. The overall dataset contained 9379 infrared images of various types of ships and their corresponding labels, including seven types of ships of different sizes: liner, bulk carrier, warship, sailboat, canoe, container ship, and fishing boat. The images in the dataset were mostly captured at nearshore ports, with a large number of ships docked in the ports, thus meeting the practical application scene requirements of the experiment. A total of 33,281 ships were collected in the dataset, with liners accounting for 4.8%; bulk carriers accounting for 7.0%; warships accounting for 9.2%; sailboats accounting for 22.8%; canoes accounting for 18.1%; container ships accounting for 2.5%; and fishing boats accounting for 35.6%. Figure 8 summarizes the number of labels for the ships in the above categories. In order to ensure the accuracy and reliability of the experimental data, we re-labeled the 977 unlabeled data and the data in the public dataset whose labels did not match the actual ship categories using the labeling annotation tool before the start of the experiments, corrected and adjusted the label formatting errors in the annotation of the public dataset, and converted the VOC format files provided in the public dataset stored in xml files into the model training required by the txt files, and converted the diagonal coordinates of the labeled box (x_min_, y_min_, x_max_, y_max_) into the form of a column of numbers with the width and height of the corresponding position. Finally, the dataset was divided into training, validation, and test sets in the ratio of 7:2:1.

### 3.2. Experimental Environment

Infrared image data acquisition equipment was InfiRay’s FT II 384 series of alarm-type long-range monitoring of infrared thermal imaging movement, the sensor was based on 12 μm ceramic detector, supported for black and white heat as well as 18 kinds of pseudo-color modes, with a resolution of 640 × 512, the frame rate of 50 Hz, and a response band of 8–14 μm.

The experiment was run on the Windows 10 operating system. The experimental machine was configured with an Intel (R) Core (TM) i7-1160G7 processor (Intel, Santa Clara, CA, USA), 16 GB of RAM, and an NVIDIA GeForce RTX 4090 (24 GB) graphics card (Nvidia, Santa Clara, CA, USA). The experimental language was python (version 3.10.8), and the deep learning framework was PyTorch (version 2.1.2).

Several key hyperparameters were set in the experiment: the number of training rounds (epochs) was 150, batch size was 16, the initial value of the learning rate (lr0) was 0.01, the optimization algorithm (optimizer) adopted was the SGD optimizer, the momentum setting (momentum) was 0.937, and the weight decay value (weight_decay) was 0.0005.

### 3.3. Evaluation Metrics

In order to demonstrate the contribution of CGSE-YOLOv5s to the performance enhancement observed in the nearshore ship detection task, we adopted commonly used performance metrics for the target detection task, including precision (P), recall (R), and mean average precision (mAP@0.5, mAP@0.5:0.95), as the evaluation metrics for the experiments. Some of our experiments also involved assessments of FPS (Frames Per Second), the model training parameters and GFLOPs (Giga Floating-Point Operations Per Second), in order to evaluate model complexity.

(12) $$Precision=\frac{TP}{TP+FP}$$

(13) $$Recall=\frac{TP}{TP+FN}$$

(14) $$mAP=\frac{\sum_{i=1}^{N}AP_{i}}{N}$$

where *TP* (True Positive) is the number of positive samples correctly identified, *FP* (False Positive) represents the number of negative samples misclassified as positive, *FN* (False Negative) denotes the number of positive samples missed, *AP* (Average Precision) is the area under the Precision–Recall curve, and *N* stands for the total number of target categories.

### 3.4. Comparison of Infrared Image Enhancement Algorithms

We used the combined strategy of CLAHE and GF algorithms to effectively resolve the problems of insufficient target texture information due to infrared imaging characteristics and excessive background noise caused by the climatic conditions of nearshore waters. In order to verify that the fusion of the two algorithms enabled our model to extract the target feature information more effectively and contributes to improvement of the model’s performance, we combined different combinations of image enhancement algorithms with the original YOLOv5s model and tested these new models separately. Meanwhile, the parameter setting of the CLAHE-GF module is an important factor that affects the detection effect of the model, so we also carried out ablation experiments with different parameter settings for the block size and the Gaussian kernel size of the CLAHE-GF to validate the detection of the dataset in this paper, and the comparison results are shown in Table 1. It is worth emphasizing that we adopted a progressive tuning strategy for CLAHE in the training phase of the improved model, adopting a stronger contrast limiting parameter clipLimit = 3.0 in the early phase of training (epoch 1–50), and gradually weakening it to clipLimit = 2.0 in the middle and late phases of training (epoch 50–150), which was designed to enable the model to be exposed to data samples with different enhancement strengths to better ensure the robustness of the model. The experimental results show that the CLAHE-GF (16, 11) fusion strategy adopted in this paper achieved higher model detection performance than any other image enhancement algorithms, which proves that the image enhancement method under the selection of this parameter is able to achieve better detection effectiveness.

### 3.5. Comparison Experiments on Attention Mechanisms

**Figure 9:**
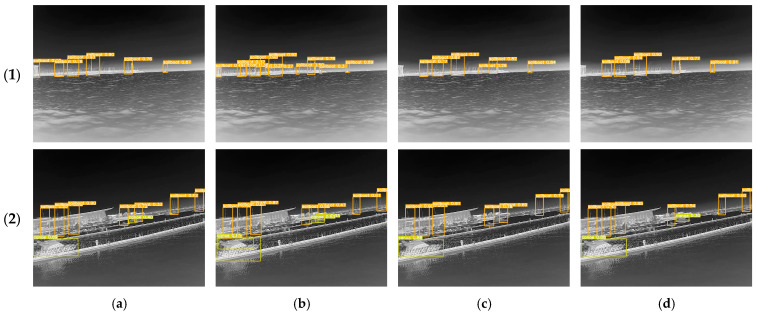
Detection results for the different attention mechanisms that were added. (**a**) YOLOv5s-SE; (**b**)YOLOv5s-EMA; (**c**)YOLOv5s-CBAM; (**d**)YOLOv5s-ECA.

In order to compare the superiority of the ECA attention mechanism used in this study with other attention mechanisms, we added four attention mechanisms (including SE, EMA, CBAM, and ECA) at the same location (i.e., in front of the detection header), and compared the mean average precision and model parameters. The results are shown in Table 2. The detection results for the different attention mechanisms that were added are shown in Figure 9.

Due to the infrared sensing characteristics, the infrared images collected in the nearshore scene will have the phenomenon that some of the ships and the background of the dock facilities overlap, resulting in a certain degree of missing ship features. The integrated ECA mechanism and the structure of the detection head adopted in this paper enable the model to reduce the focus on the background region and enhance the focus on the defective region, thus improving the model’s detection capability. In Figure 9(1), YOLOv5s-SE, YOLOv5s-EMA, and YOLOv5s-CBAM all misidentify the buildings along the shore as sailboats consistently. In contrast, YOLOv5s-ECA can avoid the misidentification between ships and objects and accurately detect the correct ship locations. In Figure 9(2), YOLOv5s-SE, YOLOv5s-EMA, and YOLOv5s-CBAM fail to accurately identify the canoe type at a relatively long distance, while YOLOv5s-ECA can distinguish the correct ship type. In addition, the results in the table show that the introduction of the ECA attention mechanism had a more significant improvement effect, in terms of mAP@0.5 and mAP@0.5:0.95, when compared to the other attention mechanisms. Therefore, the ECA attention mechanism was found to provide the optimal detection performance for nearshore infrared scenes in this study.

### 3.6. Network Structure Ablation Experiment

In order to measure the impact of the improved modules of different architectures on the model detection performance, we sequentially designed experiments by adding and replacing the improved network’s Input Layer, the feature fusion network, and the attention mechanism. The results of the ablation experiment regarding the network structure are provided in Table 3. After first introducing the CLAHE-GF data enhancement strategy, the precision of the model was improved by 0.8% but the recall did not change significantly. When the Swin Transformer module was introduced, it compensated for the lack of positive-example regression of the model and increased the recall by 1.7%; however, limited by the layer stacking design of the Swin Transformer module, the replacement of C3STR increased GFLOPs by 18.7 and decreased FPS by 50. This processing speed remained acceptable, although the increase in detection accuracy sacrifices some computational efficiency. Finally, the introduction of the ECA attention mechanism allowed the model to further take into account the channel information. The experimental results reveal that the improved model has improved the precision, recall, and mAP, with 1.2%, 2.5%, and 1.3% increases when compared to the YOLOv5s model, respectively.

### 3.7. Comparison with Other Detection Algorithms

In order to verify that the improved algorithm has relatively superior performance for the detection of ships of different categories and scales in nearshore scenarios, the algorithm was comprehensively compared with the mainstream YOLO series algorithms and other mainstream algorithms (including faster RCNN, DETR [42], both models based on ResNet50 as the backbone network) on the same dataset. The experimental results are given in Table 4.

In the YOLO series of algorithms, the YOLOv7, YOLOv8n, YOLOv8s, YOLOv10, and YOLOv12 models derived from the same baseline model were selected for comparison, and the experiments were all based on the dataset detailed earlier in this paper. Compared with other baselines of the YOLO series, the core advantage of YOLOv5s lies in its lightweight architecture and speed. Its FPS performance on the dataset of this paper is remarkable. Meanwhile, it can be seen from the table data that the detection accuracy of sailboats, canoes and fishing boats is generally lower than that of other types of ships. This is because in our dataset, these three types of ships are more numerous in complex nearshore scenes and are more difficult to detect. However, for small ship targets such as fishing boats and canoes, the performance of YOLOv5s is better than that of other baseline models of the YOLO series. Therefore, choosing YOLOv5s as the baseline of this study offered more advantages in terms of real-time performance and accuracy in nearshore target detection. As shown in Table 4, the FPS of the proposed CGSE-YOLOv5s reaches 162. Although its FPS is lower compared to some baselines, the effective balance between speed and accuracy far exceeds other baseline models. In the comparison with other series of detection algorithms, faster RCNN (R50) and DETR (R50) lag significantly behind CGSE-YOLOv5s in terms of detection speed and accuracy on the dataset used in this study. In summary, the CGSE-YOLOv5s demonstrates practical application potential in the task of infrared ship detection. By comparing the AP values of the seven types of vessels, the improved model CGSE-YOLOv5s proposed in this paper shows the most significant improvement in the detection of these several types of vessels. Moreover, its overall detection performance for almost all types of vessels is superior to that of other models. This is because the updated models such as YOLOv10 and YOLOv12 introduce more complex convolutional modules and neck designs, which require higher quality and quantity of datasets. However, due to the low image quality caused by infrared features and the complexity of nearshore scenes, the amount of data for complex scenes in the infrared nearshore is difficult to support the training of complex models, making these models unable to exert their advantages. Finally, comparing the mAP values, there were 2.1%, 1.9%, 1.4%, 1.7%, 1.2%, 7.4%, and 2.9% differences between YOLOv7, YOLOv8n, YOLOv8s, YOLOv10, YOLOv12, faster RCNN(R50) and DETR(R50) with respect to CGSE-YOLOv5s, respectively, demonstrating the excellent performance of the proposed model when applied for the detection of ships in complex nearshore scenes.

### 3.8. Comparison of Model Performance

**Figure 10:**
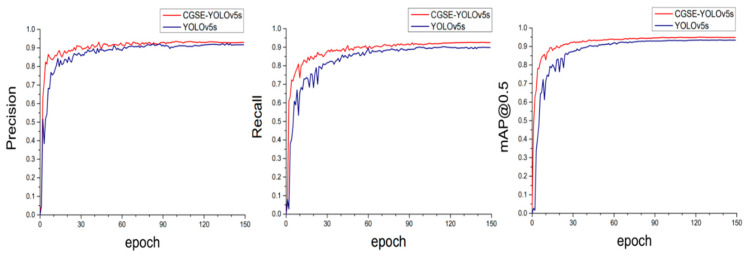
Comparison of P/R/mAP@0.5 training curves.

In order to verify the overall improvement effect of the method proposed in this paper on the model’s performance, we compared the CGSE-YOLOv5s model with the original model YOLOv5s. The comparison results are shown in Table 5. As can be seen from the data in the table, the CGSE-YOLOv5s model showed negligible change in the model parameters compared to the original model; the model complexity approximately doubled, and the detection accuracy has been obviously improved. The improvement of the detection accuracy has aggravated the computation of the improved model to a certain extent, and at the same time affected the FPS of the model. However, it can be seen through the results that, even though the processing frame rate of the new model has been compared to that of the baseline model, the FPS of the YOLOv5s has decreased, and our new model still has excellent comprehensive performance compared to other mainstream models. Meanwhile, in Figure 10, we show a comparison of the training process for the improved model (CGSE-YOLOv5s) and the original YOLOv5s model, including precision (P), recall (R), and mean average precision (mAP@0.5), using change curves. The results in the figure show that the training curves for the three indicators of CGSE-YOLOv5s quickly reached the convergence state within the first 30 epochs, gradually stabilized after 90 epochs, and finally reached a smooth state by 150 epochs, indicating that the model has good robustness.

**Figure 11:**
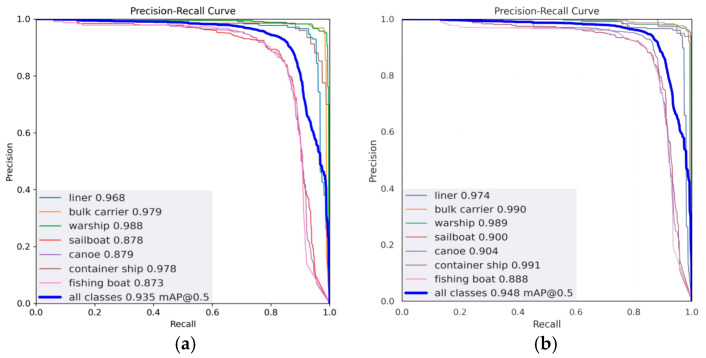
Comparison of PR curves. (**a**) YOLOv5s; (**b**) CGSE-YOLOv5s.

We plotted P–R curves to compare the performance of the original model YOLOv5s with the CGSE-YOLOv5s model (with recall as the *x*-axis and precision as the *y*-axis), as shown in Figure 11. From the P–R curve, we are able to assess the variation between precision and recall, with a larger area enclosed by a curve representing the higher performance of a model. In the nearshore multi-scale ship background, the detection of small-scale ship targets is usually more difficult. As can be seen from Figure 11a, the three types of small-scale ships—sailboat, canoe, and fishing boat, represented by the red, purple, and pink curves, respectively—had smaller areas under the curve (AUC) compared to the other ship types. Relatively, in Figure 11b, the graph surrounded by the same three curves is significantly enlarged to the upper right, which proves that the improved model has an obvious enhancement effect regarding the detection performance of small-scale ship targets. Comparison of the two models using P–R curves shows that the CGSE-YOLOv5s model outperforms the basic YOLOv5s model for nearshore vessel detection. The main improvement is reflected in higher AP values for most individual vessel categories and higher overall mAP. This suggests that improvements to CGSE-YOLOv5s can effectively improve the model’s ability to accurately detect different types of vessels in nearshore IR scenes.

### 3.9. Comparison Experiment for Visualization of Test Results

**Figure 12:**
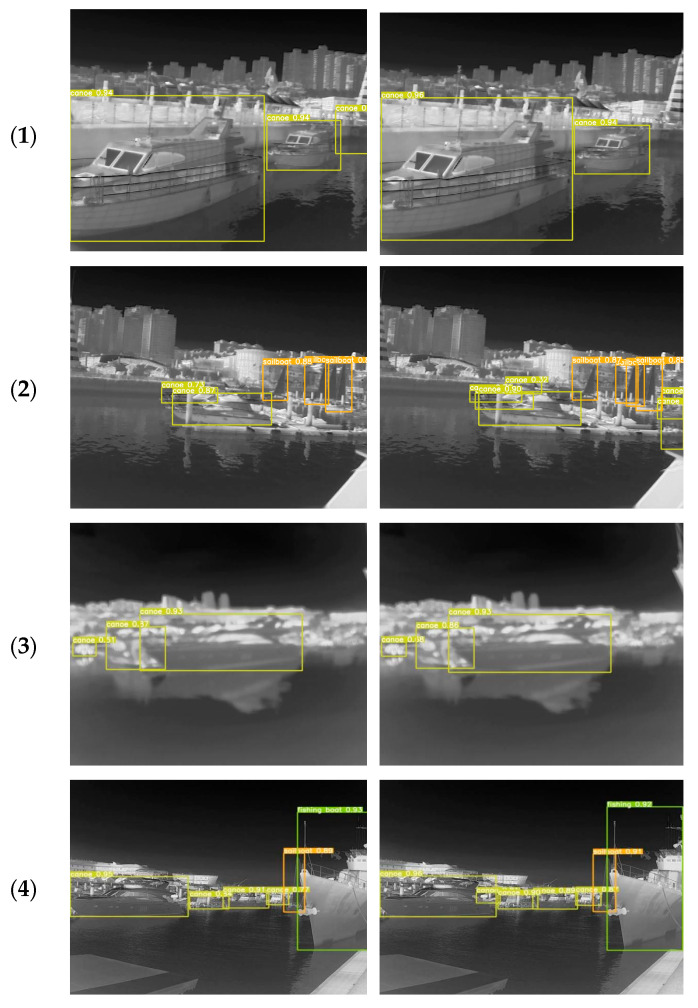

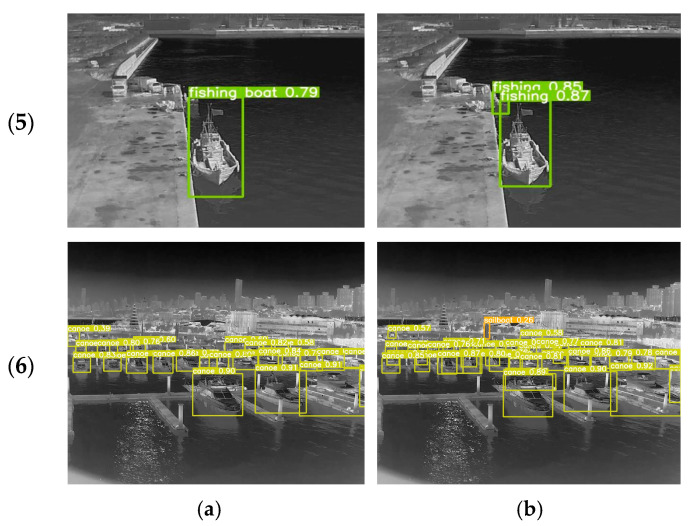
Comparison of detection results. (**a**) YOLOv5s; (**b**) CGSE-YOLOv5s.

In order to more intuitively compare the detection performance effect between the YOLOv5s and CGSE-YOLOv5s algorithms, we selected a variety of infrared nearshore scenes with complex backgrounds and other interference problems for visualization and comparison. The detection results of the two algorithms are shown in Figure 12, where (a) is the detection result of YOLOv5s and (b) is the detection result of CGSE-YOLOv5s.

The figure shows the ship distributions in nearshore water scenes presenting complex scenarios such as dense, multi-scale objects, mutual occlusion, insufficient image resolution, and interference from rear buildings. The comparison results show that the original YOLOv5s algorithm has difficulty in detecting small and occluded targets in complex nearshore water scenes, often leading to target loss and false detection. In contrast, the CGSE-YOLOv5s algorithm effectively suppresses background interference in different nearshore scenarios, enabling it not only to identify multiple ship targets that were undetected or falsely detected by the original model, but also to achieve high confidence results. Our algorithm adopts the CLAHE-GF traditional image enhancement fusion strategy to refine the edge feature information of infrared images, accurately identify targets of different scales by integrating C3STR to capture multi-scale features, and enhance the ability to capture ship features through the ECA mechanism, thereby providing excellent performance for ship detection. Comprehensive comparative analysis verified that the CGSE-YOLOv5s algorithm proposed in this study can effectively reduce the false detection rate and omission rate of infrared ship targets in complex nearshore scenes, providing excellent performance in terms of detection accuracy.

## 4. Discussion

The CGSE-YOLOv5s model proposed in this paper can be applied to target detection tasks involving images of ships in nearshore waters with complex backgrounds, while maintaining excellent detection performance (including accuracy and speed). CGSE-YOLOv5s synthesizes image enhancement operations and improved convolution neural network structures, unlike other research which has involved making direct improvements only to the network structure. The proposed algorithm prioritizes the quality of the infrared images, aiming to improve the contrast between the target and the background within the image and reduce the effects of low resolution for optimization of the network structure. CGSE-YOLOv5s includes the Swin Transformer module to extract more adequate global information based on the model’s self-attention mechanism. Due to the general application of attention mechanisms and their role in the performance of the network, we tested a variety of attention mechanisms and compared the differences between them and the ECA mechanism; in this way, we found the ECA mechanism to be more suitable for targeting the infrared scene, as it obtained superior results when compared to several other mechanisms. Due to the superior detection performance demonstrated by the proposed CGSE-YOLOv5s, compared to other models, the developed technology expands the potential of ship detection applications characterized by complex nearshore scenarios.

However, despite the considerable improvement in detection accuracy in nearshore scenarios achieved by the proposed model, we did not conduct in-depth research on similar tasks in different scenarios, such as the detection of small targets at long distances. As such, we will explore more effective methods to conduct further research on various ship detection scenarios in the future. In addition, although infrared image enhancement based on traditional algorithms can achieve certain results, there are still some limitations, such as poor generalization in cross-dataset scenarios, and in the future, we will apply the latest neural network-based image enhancement mechanism considering model lightweight requirements in order to train the model more effectively.

## 5. Conclusions

Considering the problem regarding the limited ship target detection effect in infrared images of complex nearshore scenes, we proposed an improved infrared ship detection method based on YOLOv5s. The proposed method designs a traditional image enhancement strategy combining CLAHE and GF, aiming to improve the quality of infrared images and enrich the texture information of the target; the integration of Swin Transformer coding structure in the feature pyramid network enhances the model’s ability to extract the global feature information, which effectively responds to the challenge of detecting the significant differences of the target’s multiscale differences. In order to strengthen the network’s attention to the key information of infrared ships, an ECA mechanism is introduced to improve the capture of target features. Our experimental results under the infrared nearshore scene dataset revealed the mean average precision of the proposed CGSE-YOLOv5s algorithm, when applied to nearshore water scenes, was 94.8%, an improvement over the 93.5% achieved by YOLOv5s, with a 1.2% increase in precision and a 2.5% increase in recall, thus, effectively reducing the false and misdetection rates of ships in the complex nearshore scenes while improving the classification of such ships. Meanwhile, by comparing it with other mainstream detection algorithms, the detection algorithm in this paper shows more significant performance advantages when applied to nearshore infrared ship detection. These results reinforce the practical significance of this paper’s contributions. In the future, we aim to cooperate with more research organizations to obtain more small, long-distance infrared ship data types, allowing the dataset used in this study to be expanded and, thus, the performance of the model presented in this paper to be further improved. Additionally, we will explore the application of more efficient lightweight detection models in real engineering deployments.

## Figures and Tables

**Table 1:** Comparison results for image enhancement algorithms.

	mAP@0.5/%	mAP@0.5:0.95/%
AHE-YOLOv5s	92.9	61.6
CLAHE-YOLOv5s (16)	93.7	63.3
GF-YOLOv5s (11)	93.5	61.9
YOLOv5s + CLAHE + GF (8, 9)	93.5	62.3
YOLOv5s + CLAHE + GF (8, 11)	93.6	62.6
YOLOv5s + CLAHE + GF (16, 9)	93.9	63.5
YOLOv5s + CLAHE + GF (16, 11)	**94.1**	**64.4**

The top-performing data are highlighted in bold.

**Table 2:** Comparison results for the different attention mechanisms that were added.

	mAP@0.5/%	mAP@0.5:0.95/%	Params/M	GFLOPs
YOLOv5s-SE	93.5	62.7	7.1	17.5
YOLOv5s-EMA	93.3	61.5	7.0	16.0
YOLOv5s-CBAM	93.5	62.1	7.4	26.4
YOLOv5s-ECA	**93.8**	**63.4**	7.1	16.3

The top-performing data are highlighted in bold.

**Table 3:** Results of ablation experiment.

Model	Precision/%	Recall/%	mAP@0.5/%	GFLOPs	FPS
YOLOv5s	91.7	89.8	93.5	15.8	238
CLGF-YOLOv5s	92.5	89.8	94.1	16.0	224
CLGF-YOLOV5s-STR	92.5	91.6	94.5	34.7	174
CLGF-YOLOV5s-STR-ECA	**92.9**	**92.3**	**94.8**	35.6	162

The top-performing data are highlighted in bold.

**Table 4:** Comparison with other mainstream algorithms.

Model	Average Precision (AP)/%							mAP@0.5/%	FPS
	Liner	Bulk	Warship	Sailboat	Canoe	Container	Fishing		
Faster RCNN (R50)	86.7	90.7	94.6	82.4	83.3	94.3	79.8	87.4	54
DETR(R50)	88.9	95.4	98.7	88.7	86.6	96.7	88.3	91.9	95
YOLOv5s	96.8	97.9	98.8	87.8	87.9	97.8	87.3	93.5	238
YOLOv7	95.5	97.8	98.6	86.9	87.1	97.2	85.9	92.7	240
YOLOv8n	96.9	97.9	98.8	86.7	86.9	97.9	85.5	92.9	221
YOLOv8s	95.9	**99.1**	98.3	88.0	87.4	98.1	87.2	93.4	184
YOLOv10	96.5	98.4	98.8	88.1	86.3	97.5	86.2	93.1	102
YOLOv12	96.7	98.3	**99.2**	88.0	87.6	98.6	86.7	93.6	151
CGSE-YOLOv5s	**97.4**	99.0	98.9	**90.0**	**90.4**	**99.1**	**88.8**	**94.8**	162

The top-performing data are highlighted in bold.

**Table 5:** Comparative results of model performance.

	YOLOv5s	CGSE-YOLOv5s
Precision/%	91.7	**92.9**
Recall/%	89.8	**92.3**
mAP@0.5/%	93.5	**94.8**
mAP@0.5:0.95/%	61.4	**66.7**
Params/M	7.0	7.2
GFLOPs	15.8	35.6
FPS	238	162

The top-performing data are highlighted in bold.

## Data Availability

The data in this study are included in the article.
